# Optimised padlock probe ligation and microarray detection of multiple (non-authorised) GMOs in a single reaction

**DOI:** 10.1186/1471-2164-9-584

**Published:** 2008-12-04

**Authors:** Theo W Prins, Jeroen P van Dijk, Henriek G Beenen, AM Angeline Van Hoef, Marleen M Voorhuijzen, Cor D Schoen, Henk JM Aarts, Esther J Kok

**Affiliations:** 1 RIKILT – Institute of Food Safety (WUR), Bornsesteeg 45, 6708 PD Wageningen, the Netherlands; 2 Plant Research International BV (WUR), Droevendaalsesteeg 1, 6708 PB Wageningen, the Netherlands

## Abstract

**Background:**

To maintain EU GMO regulations, producers of new GM crop varieties need to supply an event-specific method for the new variety. As a result methods are nowadays available for EU-authorised genetically modified organisms (GMOs), but only to a limited extent for EU-non-authorised GMOs (NAGs). In the last decade the diversity of genetically modified (GM) ingredients in food and feed has increased significantly. As a result of this increase GMO laboratories currently need to apply many different methods to establish to potential presence of NAGs in raw materials and complex derived products.

**Results:**

In this paper we present an innovative method for detecting (approved) GMOs as well as the potential presence of NAGs in complex DNA samples containing different crop species. An optimised protocol has been developed for padlock probe ligation in combination with microarray detection (PPLMD) that can easily be scaled up. Linear padlock probes targeted against GMO-events, -elements and -species have been developed that can hybridise to their genomic target DNA and are visualised using microarray hybridisation.

In a tenplex PPLMD experiment, different genomic targets in Roundup-Ready soya, MON1445 cotton and Bt176 maize were detected down to at least 1%. In single experiments, the targets were detected down to 0.1%, i.e. comparable to standard qPCR.

**Conclusion:**

Compared to currently available methods this is a significant step forward towards multiplex detection in complex raw materials and derived products. It is shown that the PPLMD approach is suitable for large-scale detection of GMOs in real-life samples and provides the possibility to detect and/or identify NAGs that would otherwise remain undetected.

## Background

In recent years there have been a number of incidents in which not (yet) EU-approved GMO varieties were present in shipments imported into the EU. Examples are Bt10 maize [1], LL601 rice [2] and Bt63 rice [3]. As a result the EU has formulated additional regulations in the case of the Bt10 maize and Bt63 and LL601 rice varieties that stipulate that in specific shipments it needs to be certified that the named unapproved varieties are not present [1-3]. With the increasing complexity of world trade networks, asynchronous approval of GMO varieties in different parts of the world leads to the increased possibility that non-authorised GMOs (NAGs) are present in EU import batches.

**Figure 1:**
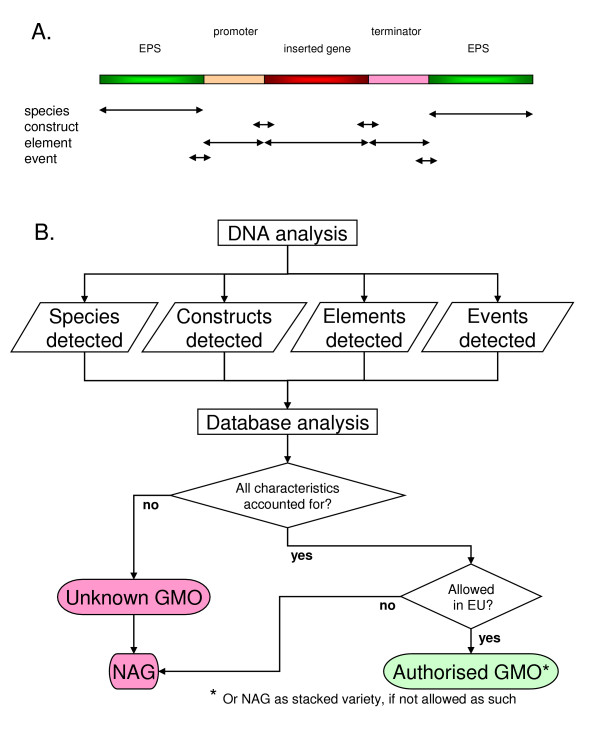
**Padlock probe locations in a GMO and GMO decision tree**. Scheme for the location of specific padlock probes on species-, construct-, element- and event level of a GMO. EPS = endogenous plant sequence (A). GMO decision tree in which the outcomes of DNA analyses are matched with the data from a database on known GM crop events to elucidate the presence and status of GMOs in a test sample. Here, events, elements, constructs and species are detected and compared with the database information. Detection of unknown GMOs is by exclusion of profiles of known GMOs present within the sample (B).

Producers of new GM crop varieties to be marketed within the EU need to supply an event-specific method for the new variety as well as the related positive and negative reference materials [4,5]. As a result methods are nowadays available for EU-authorised genetically modified organisms (GMOs). On the other hand, methods for NAGs are only available to a very limited extent. In order to maintain current EU GMO regulations it is necessary to check for the presence of NAGs on the basis of available methods for GMO-events, -elements (or -constructs i.e. bridging elements) and -species according to the scheme as presented in Figure 1, leading to an increasing number of analyses per sample and shipment. This scheme uses available information on approved GMO crop varieties as a basis for the detection of the presence of authorised GMOs, NAGs and possibly even unknown GMO varieties. Therefore, multiplex methods need to be developed to cover diversification of GMOs, both authorized and NAGs.

Effective multiplex methods for GMO detection and identification are being developed to reduce time and associated costs of analysis. This is one of the main aims of the EU integrated project Co-Extra: the development of a multiplex method for GMO analysis that can screen an individual sample for the presence of a large number of GMO varieties and GMO elements in a single analysis. Different multiplex approaches have already been described in the scientific literature for GMO detection and identification [6]. Initially multiplex PCR was the method of choice. Sensitive detection of eight maize GMO events is described by Heide *et al. *[7] with qualitative multiplex PCR and fluorescence capillary electrophoresis (CE) detection with a detection limit of 0.1%. The method was optimised for samples containing 100% maize of which 0.4–0.6% was of GMO origin. An undefined negative sample was included. The application of differential quantitative PCR (dQ-PCR: [8]) was a step in detecting NAGs by means of a statistical model. Here, an event-specific PCR is compared quantitatively with a PCR for P35S and presence of NAGs is inferred when the number of molecules differs significantly from the expected number in the known event. Demeke and Ratnayaka [9] developed a multiplex PCR assay for the detection of GM-canola OXY235 and T45 in canola samples with a detection limit of 0.1%. The assay was also shown to be valid when traces were present in a wheat or barley background.

Others combined PCR analysis with microarray detection. By using regular PCR in combination with microarray detection, Xu *et al*. [10] were able to multiplex up to three primer pairs in one reaction with a single GMO as target. The false positives (i.e. CaMV T35S and *nos *promoter in RRS) and absence of negative controls do suggest that the method could be further optimised. Multiplex PCR amplification and detection by low-density microarray was performed by Zhou *et al*. [11] to detect target elements in Roundup-Ready soya (5) and Ms1/Rf1 canola (6). Multiple genes were detected using Cy5-dUTP within one GMO and detection was shown down to 0.5% when optimised primer concentrations were used per GMO. No signal was observed in the negative control, but it should be noted that this control comprised 50% DMSO. In a collaborative trial, Leimanis *et al*. [12] applied microarrays for the detection of preamplified multiplex PCR products of GMO samples (9 GMOs, 5 plant species and 3 elements) with biotinylated primers, reaching sensitivity down to 0.1%. Due to the low detection level, the authors state that false positive signals may occur and that complex samples may not be successfully analysed by the method. Nevertheless this is the first validated multiplex study for GMO detection using an array format.

In some cases microarray detection was used without initial amplification step. In Nesvold *et al*. [13] and Tengs *et al*. [14] a microarray was applied containing 25-basepair probes from 235 different vector sequences to detect (unknown) GMOs using statistical probability calculations. Although the method is very efficient in detecting vector sequences, false positives may occur when no true positive sample is present.

Other approaches were tested as well. Multidetection of viral DNA was investigated by Bouchet *et al*. [15] using electrochemical detection of cylinder-shaped conducting polypyrrole. Here, a biochip was used to detect synthetic viral DNA targets with detection limit of 100 pM. The labelless approach was performed with two targets and absence of DNA as negative sample, although genomic DNA of a 100% GMO resulted in a much lower signal compared to the synthetic target. An electrochemiluminescence-based simplex bio-barcode method [16] used gold nanoparticles, barcoded DNA in combination with streptavidine-coated magnetic beads and an electrode for detection. Quantitative analysis using digested and purified gDNA allowed detection, but not yet on multiple targets or in complex genomic samples.

In recent years another promising approach has been tested by a number of research groups. This approach combines a ligation reaction, subsequent amplification and (microarray) detection [17]. Peano *et al*. [18] analysed for the presence of two species (comprising 5 different GMOs) on the basis of 7 primer combinations. Their technique makes use of PCR amplification and a subsequently a linear ligation detection reaction, followed by detection of Cy3-signals on a universal array containing ZIP-codes. This method has a sensitivity of 50 molecules in maize and 100 molecules in soya. Pang *et al*. [19] investigated the use of rolling circle amplification for the detection of genetically modified food. Here a pre-amplification PCR step was performed prior to the ligation reaction, rolling circle amplification and subsequent PCR amplification. Detection of 5% MON810 maize was possible by gel electrophoresis, although the authors anticipated difficulties in the extension of the method. Ehlert *et al*. [20] used ligation dependent probe amplification in combination with CE-detection. Synthetic targets (canola) and genomic DNA (Roundup-Ready soya (RRS) and MON810) could be detected at 5%, while RRS event and CaMV P35S could be detected in 0.1% RRS. A difficulty that has often been encountered with this approach is the presence of (too high) background values for negative samples.

These publications on the combination of ligation, amplification and microarray detection show the potential of this approach. Also in other areas this strategy has already shown its value [21,22].

In the present study ten different padlock probes were tested in a multiplex setting according to an optimised padlock probe ligation – microarray detection (PPLMD) strategy [23]. In short, a linear padlock probe with 5' and 3' target sequences can hybridise to their genomic counterpart. Upon hybridisation, the juxtaposed ends are ligated to form a circular molecule. The circular molecules are amplified by PCR with a universal forward and reverse-Cy3 primer. Each probe contains a unique DNA sequence (ZIP-code). Large amounts of linear ssDNA with a Cy3-labelled cZIP code are generated, which is visualised after hybridisation on a microarray.

The padlock probes used in the present study included four plant species probes, two GMO event-specific probes, three GMO element-specific probes (Figure 1) and one control probe for an artificial template. The probes were tested in dilution series ranging from 0.1 to 5% in simplex experiments, and on a mixture of GMOs in different percentages. The optimized PPLMD procedure presented here has significantly reduced background values compared to similar approaches as documented in the scientific literature, thereby increasing the sensitivity of the approach up to the levels required for the maintenance of EU GMO regulations. Furthermore, this is the first series of experiments using real-life complex samples with total genomic DNA, demonstrating the practical applicability of the PPLMD strategy for the detection of GMOs and NAGs, and thus for the maintenance of (EU) GMO regulations.

## Methods

### Plant materials and mixtures

See Table 1 for overview of wild type and GMO plant material. For detailed information on the composition of the GMOs used, see GMO Detection method Database (GMDD: [24,25]), AGBIOS [26] or the RIKILT GMO portal [27].

**Table 1:** Plant material for DNA isolation with catalogue codes of reference standards (w/w).

**Plant material**	**code**	**Plant material**		**code**
RRS 0.0%	ERM-BF410a	MON1445	0%	AOCS 0804-A
RRS 0.1%	ERM-BF410b	MON1445	100%	AOCS 0804-B
RRS 0.5%	ERM-BF410c			
RRS 1.0%	ERM-BF410d	canola	0%	AOCS 0304A
RRS 5.0%	ERM-BF410f	rice	0%	Indica (Xieqingzao)
Bt176 0.0%	ERM-BF411a	sugar beet	0%	ERM-BF419a
Bt176 0.1%	ERM-BF411b	potato	0%	ERM-BF421a
Bt176 0.5%	ERM-BF411c			
Bt176 1.0%	ERM-BF411d	MON810	100%	RIKILT collection
Bt176 5.0%	ERM-BF411f	RRS	100%	RIKILT collection

Ground seed material was purchased from IRMM (Geel, Belgium: RRS soya, Bt176 maize, sugar beet and potato) and AOCS (Urbana, IL, USA: MON1445 cotton and canola); see Table 1 for details. For MON1445, no commercial mixtures were available, so these were composed by mixing DNA isolated from the commercial reference standards 0 and 100% MON1445. 176 = SYN-EV176-9 or Bt176; GTS 40-3-2 = MON-Ø4Ø32-6 or RRS; MON1445/1698 = MON-Ø1445-2 or MON1445.

For non-GMO samples, species were selected on the basis of divergent background to broaden the target gene pool. DNA was isolated from potato, sugar beet, canola and rice and mixed in equal amounts (w/w).

With the haploid genome weight (RBG Kew Plant DNA C-values database: [28]), the C-value reflects the weight of a haploid genome in pg. Thus, the calculated number of targets within a haploid genome in 200 ng is: cotton (*Gossypium hirsutum*) = 61,920, which is set to 1; maize (*Zea mays*) = 73,260, which is 1.18 relative to cotton; canola (*Brassica napus*) = 173,913, which is 2.81 relative to cotton and soya (*Glycine max*) = 176,991, which is 2.86 relative to cotton.

### DNA extraction

For DNA isolation of all samples, 100 mg material was used per isolation. For DNA isolation the next protocol was used for all samples but RRS: 100 mg plant material, 150 μl MilliQ treated water (MQ) and 350 μl CTAB buffer (20 g/l CTAB; 1.4 M NaCl; 0.1 M Tris-HCl; 20 mM EDTA) was mixed together with 5 μl RNaseA (Qiagen) and incubated 15 min at 65°C. Then 20 μl 20 mg/ml Proteinase K (Fermentas Molecular Biology, Germany) was added and incubated 15 min at 65°C. Buffer AP2 (200 μl, Qiagen DNeasy Plant Minikit) was added and this was incubated on ice for 5 min. Further steps continued from step 10 of the Qiagen DNeasy Plant Minikit as described by the manufacturer's protocol (Qiagen: DNeasy plant handbook 07/2006) without modifications. DNA concentrations were measured with the NanoDrop spectrophotometer (NanoDrop ND-1000, V3.5.2). DNA from RRS material (100 mg) was isolated using protocol 07/2006 of the Qiagen DNeasy Plant Minikit.

### Ligation detection probes

BLAST analysis [29] was performed with the gDNA target sequence to verify the specificity through the lack of homology with other crops/cultivars. It should be noted that not all genome sequences are in the database and only verification by experimentation can validate the padlock probe as crop/cultivar specific.

Requirements to have a ~30 nt 5' target with a T_m _of 68–70°C and a ~15 nt 3' target with a T_m _of 40°C were fulfilled. The T_m _calculations were performed using HYTHER™ (version 1.0, Nicolas Peyret and John SantaLucia, Wayne State University; [30-32]). Mfold [33] was used to optimise the design to eliminate significant secondary structures in the molecule. The length of the designed padlock probes varied slightly since the target sequences had to meet the requirements.

The padlock probes all contain a 5' phosphate group to allow ligation. See Table 2 for used sequences and cZIP-codes. Concentration of the padlock probes (Biolegio, NL) were measured with the NanoDrop spectrophotometer. A stock was prepared containing a mixture of probes in a concentration of 250 pM each. The ten selected padlocks were mixed to 12.5 nM and this mixture was used in the experiments.

**Table 2:** ssDNA sequences of the oligonucleotides used in padlocks, target molecules and primers (5'-3' orientation).

**Name**	**5' target**	**cZIP sequence**	**3' target**	**Target gene (GenBank:)**	**Size (nt)**
Cotton sp.	CTGGGCTGAGAACAACATTCTGACTCACCTCAAACCA	AAGTGTGCCAGACGCTCGAA	CTTTAAATCTTTGGAGGG	[AJ132636] (sad1)	122
Maize sp.	CTGTGGCATCATCACTGGCATCGT	GTACTACATTCGTGCGATGG	TTAGGCGTCATCAT	[AF371266] (zein)	124
Canola sp.	GTGACGCATACGTTCTATAACATCAGCCTGTCC	CGTCGCGTTAGACAGCTCAT	CCGATCTTTCTTGTATTC	[DQ173668] (ACCase)	118
Soya sp.	GCATCATAGGTAATGAGAACCTTGGCTACTTTATTGTTGGCC	ACTCCAGTGCCAAGTACGAT	AGAGGCTGGTGGAG	[K00821] (lectin1)	142
RRS event	GATCCCAAATAGTTTTGTTTTTCTAACAACGAGAAGCTATATGTAGATGCTATT	AACAACGATGAGACCGGGCT	TCAAACAGTTCTTCTCC	[AJ308515]	138
MON810	GGCAATGGCAAAGGATGTTAAACGTTAGAGTCCTTCGT	TGCCCTATTGTTGCGTCGGA	AAAGTGACAGATAGCTG	[AF434709]	141
Bt176 event	GAACATCAGATCTCGGTGACGGGCAGGACC	TAATCTAATTCTGGTCGCGG	GCATCAATGGAGGAGA	[AJ878607]	113
CaMV P35S	CGAAGGATAGTGGGATTGTGCGTCATCCCTTACG	CCACGAGCTGTAATCCGGTA	ATAGAGGAAGGGTCTTG	[V00141]	118
FMV P35S	TCTTCGGTGGATGTCTTTTTCTGAAACTTACTGACCATGATG	GTGATTAAGTCTGCTTCGGC	GCCCACTAACTTTAAG	[X06166]	125
bar	TCGATGTAGTGGTTGACGATGGTGCAGACCG	CGAGTGCTCCGTGCGAAATA	TGACCGTGCTTGTC	[AY346130]	112
SpikeLock	CGTCGGACAGGTTACTTTCGAAGAGCCGGAATACTC	GCTGAGGTCGATGCTGAGGTCGCA (cZIP-P for Positioning)	CGAAGGTCATATCTCG		123
ZB3		CGTGCAAGTTACCGAGCTGA			20
	**primer sequence**				
Fwd primer	GCAAGAGATGGGCTACAGAGGAT				23
Fwd25 primer	CCGCAAGAGATGGGCTACAGAGGAT				25
Rev primer	GGACAGACACGCTAAGACAGAACT				24

To test padlock probes, artificial target sequences (Biolegio, NL) were used. These are single stranded target sequences complementary to the combined 5' and 3' target sequence of the padlock probe to allow hybridisation and subsequent ligation of the padlock probes on the juxtaposed 5' and 3' target.

### Ligation

Four μl DNA (200 ng of either single DNA or mixed DNA sample) was used in a ligation assay (1× *Pfu *ligation buffer (Stratagene); 12% PEG6000 (Fluka, Germany); 0.1 U/μl *Pfu *ligase (Stratagene), 1.0 pM SpikeTarget and 25 pM of each padlock probe in a final volume of 10 μl) to allow circularisation (94°C for 5 min; 95°C 30 s, 65°C 5 min for 30 cycles) in the BioRad iCycler 3.021.

Ligated padlock probes were subsequently analysed in real-time PCR or labelled and visualised on microarray.

### Real-time PCR

Real-time PCR was performed using SYBR Green (1× SYBR Green Supermix containing the hotstart iTaq DNA polymerase (BioRad); 0.5 μM forward primer; 0.5 μM reverse primer (Biolegio, NL); 3 μl ligation mixture) in the BioRad iQ5 multi-colour Real-time Detection System (95°C for 3 min; 95°C 10 s, 60°C 45 s for 40 cycles; 95°C 1 min). The melting curve was monitored from 55 to 95°C in 80 steps of 0.5°C per 10 s. The data were analysed using the optical system software program iQ5 version 2.0 (BioRad). See Table 2 for the sequence of the forward and reverse primer.

The threshold cycle (C_t_) represents the PCR cycle at which a noticeable increase in SYBR Green™ fluorescence above a baseline signal is first detected. ΔC_t _is the difference between the C_t _of a sample assay and the C_t _of the no template control: ΔC_t _= C_t(target) _- C_t(MQ)_.

### Padlock probe performance testing

To confirm specificity of every single padlock probe, 25 pM of the padlock probe was tested on artificial target and on 200 ng genomic target DNA. A ligation was performed on a dilution range of artificial target (0.1–1 pM) to prove that the padlocks indeed recognise their target.

### Microarray visualisation

Slides with two microarrays each were ordered from Isogen (NL) and contained 100 spotted ZIP-codes (20-mer oligonucleotide sequences from Affymetrix) with a 10-mer A-tail (and C6 to linker) in quadruplicate per microarray. The ZIP-codes are demarcated by ZIP-P spots (Table 2) for positioning purposes.

The microarrays were pre-hybridised for 75 min at 42°C in previously boiled and rapidly cooled pre-hybridisation mix (5× SSC; 0.1% SDS; 0.1 mg/ml herring sperm DNA (Madison WI, USA)). After a repeated wash with 0.1× SSC for 5 min at RT on a rotary tablet, the slides were rinsed with MQ and dried by centrifugation (2 min at 1000 rpm).

For probe amplification, 4 μl ligation mixture was amplified using the forward25 and reverse primer (see Table 2) sites on the padlock probe (1× ThermoPol PCR buffer (New England Biolabs, containing 2 mM MgCl_2_); 2.5 mM MgCl_2_; 200 μM dNTPs; 2 U Vent^® ^exo^- ^DNA polymerase (New England Biolabs); 500 nM Cy3-labelled reverse primer; 50 nM forward25 primer in a total volume of 25 μl). After Linear After The Exponential (LATE)-PCR amplification (95°C for 15 min; 95°C 15 s, 51°C 2 s, 72°C 5 s for 80 cycles), using an asymmetrical primer concentration of which the primer present in the lower concentration is corrected for T_m _by increasing the length of the primer, 2 μl denatured labelled mix was applied to 63 μl hybridisation mixture (5× SSC; 0.1% SDS; 0.1 mg herring sperm DNA; 192 pM 5'Cy-labelled cZIP mix) to hybridise to the microarray in a confined area (Gene Frame frames and cover slips 1.5 × 1.6 cm for 65 μl, ABgene, UK) for 2 h at 65°C in a moist atmosphere (MJ Research, PTC-200 thermo cycler with Alpha Unit™ block assembly). 5'Cy-labelled cZIP mix was prepared by adding Cy5-labelled cZIP targets of the ZIP-codes used in this experiment in a final concentration of 12.5 nM to allow internal normalisation per spot based on the Cy3 and Cy5 signal ratio. Cy3- and Cy5-labelled cZIP-B3 was used as a hybridisation control.

After removing the chambers the microarrays were washed twice in 1× SSC; 0.1% SDS for 5 min, twice in 0.1× SSC; 0.1% SDS for 5 min, twice in 0.1× SSC for 1 min and once with 0.01× SSC for 30 s on a rotary tablet. The slides were dried by centrifugation (2 min at 1000 rpm) and stored in the dark.

The ScanArray Express HT microarray scanner (Perkin Elmer) was used to scan the signal of the individual spots at 543 nm (Cy3) and 633 nm (Cy5). Here, a Photo Multiplier Tube (PMT) gain was used between 55 and 70 at a laser power of 90%. The individual signals were quantified using the optical system software program ArrayVision version 8.0 (Imaging Research Inc.) and processed in Microsoft Office Excel 2003.

### Data analyses

The signal to noise ratio (S/N) was defined as the spot signal minus the background signal, divided by the standard deviation of the background signal. Values with an S/N ≤ 3 were classified as negative signal. Outliers and obvious artefacts were removed manually.

Data points were normalised according to [(Cy3/Cy5_target_)/average(Cy3/Cy5_P_)]×1000. Here, P is signal from the positioning spot generated by the SpikeLock padlock and the artificial SpikeTarget, which was used to normalise the target signal. For GMOs, each sample was hybridised in duplicate generating a maximum of 8 signals per target. For P, the number of signals was 32.

Sensitivity was established by performing a one-tail students' t-test on normalised signals from a positive sample and a negative sample. For the t-test all values were used except obvious outliers, to prevent a bias in the t-test from application of the S/N filter. A *p*-value < 0.05 was interpreted as the 'positive' signal being significantly higher than the 'negative' signal.

## Results

### Optimisation

**Figure 2:**
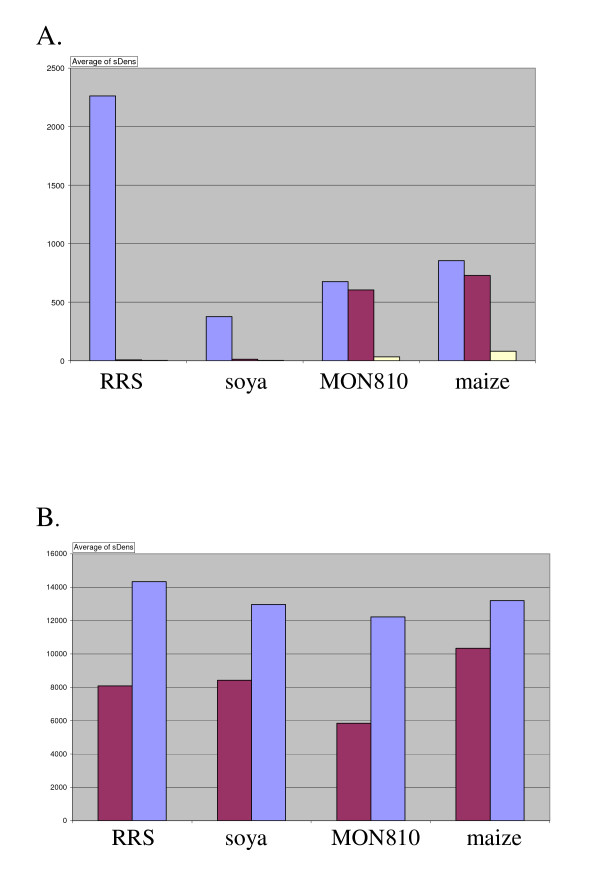
**Optimisation of the PPLMD method**. The y-axis represents the average background-subtracted pixel density (0–64,000). A. Introduction of SpikeTarget DNA reduced background in negative control (purple vs. yellow). Blue: genomic DNA (containing RRS and MON810) + SpikeTarget, purple: MQ, yellow: MQ with SpikeTarget. All 4 padlock probes were present in the mix. HotStar Taq DNA polymerase was used to amplify in 40 LATE cycles. B. 80 LATE cycles increased signal compared to 40 cycles. Purple: 40 LATE cycles, blue: 80 LATE cycles. All 4 padlock probes were present in the mix (containing RRS and MON810). Vent^® ^exo^- ^DNA polymerase was used.

Spiking the reaction with a known amount of synthetic target (SpikeTarget) onto which a SpikeLock could circularise, improved the results significantly. The reaction that is now part of the standard procedure functioned as ligation control and positioned the microarray (as a result of the choice for ZIP-P (positioning) that was previously added to the reaction as Cy3-labelled cZIP-P oligonucleotide) and prevented nonspecific padlock reactions when no specific target was present (Figure 2A).

The initial standard protocol with HotStar Taq DNA polymerase (Qiagen) as to amplify the circular padlock probe was altered by application of the 5'-3' exonuclease-deficient Vent^® ^exo^- ^DNA polymerase, theoretically allowing extended linear amplification of the circular padlock target molecule and subsequent logarithmic PCR amplification. The results showed a tendency that positive signals were improved and background signals were decreased compared to the use of HotStar Taq DNA polymerase, although statistical evidence is lacking (results not shown).

In previous experiments [23] amplification of the circular padlock was performed with asymmetrical PCR using a limiting concentration of the forward primer. This allowed exhaustion of the forward primer, switching from logarithmic amplification into linear amplification with the Cy3-labelled reverse primer. The PCR amplification was performed with 40 cycles. The amplification of labelled molecules was improved by increasing number of LATE cycles from 40 to 80 cycles and by extending the forward primer to compensate for the lowered T_m _due to the lower concentration (Figure 2B).

Prior to hybridisation on the microarray, the probe was denaturated to prevent double stranded labelled molecules that could reduce efficient hybridisation onto the microarray.

The combination of these adjustments to the protocol resulted in a better signal on the microarray, less background and an internal ligation control that functioned as a positioning spot as well.

### Validation of padlock probes

Padlocks were validated in two ways: 1) their capacity to circularise on their artificial target, 2) their performance on genomic DNA. In all cases, the padlock probes showed a significantly lower C_t _value in real-time qPCR for the artificial target than for the non target control, indicating a more specific hybridisation. Furthermore, melting curve analyses suggested that the signals found in the water samples were more probably due to primer-dimer like artefacts than specific (background) amplification (not shown). Next, the padlock probes were tested on genomic target DNA (100% GMO when available). These qPCRs resulted in comparable C_t _values compared to synthetic DNA.

For microarray analysis, each padlock probe was tested in simplex and multiplex to establish the sensitivity and to screen for possible cross-hybridisation with other ZIP-codes on the array. Cross-hybridization between the Cy3-labelled (cZIP on the) padlock probe and other ZIP-codes that were used on the microarray was not observed (results not shown).

### Performance in simplex GMO experiments

The sensitivity (i.e. the smallest concentration of a target analyte that can be determined and is distinguishable from a zero result; *p *< 0.05) of the probes was tested by comparing the signals in a 0% sample to an increasing amount of GMO (0.1, 0.5, 1 and 5%, see Table 3). It was shown that in only two cases the sensitivity of the PPLMD approach in simplex settings was 5%, in all other cases the sensitivity was 0.5% or even 0.1%.

**Table 3:** Sensitivity of GM padlock probes.

	**Bt176**	**MON1445**	**RRS**	**GM mix**
**CaMV P35S**	0.1%	0.5%	0.1%	1.0%
**FMV P35S**	**n.p.**	5.0%	**n.p.**	1.7%
***bar***	0.1% (0.5*)	**n.p.**	**n.p.**	1.7%
**Bt176 event**	0.5%	**n.p.**	**n.p.**	2.5%
**RRS event**	**n.p.**	**n.p.**	5.0%	2.5%

Bt176, MON1445 and RRS performed as simplex, GM mix performed as multiplex.

*level of sensitivity when all 'negative' samples were included in the analysis.

n.p.: not present.

1.0% GM mix consists of 20% maize, soya and cotton (5% GMO each) and 40% canola.

1.7% GM mix consists of 33% maize, soya and cotton (5% GMO each).

2.5% GM mix consists of 50% maize and soya (5% GMO each).

**Figure 3:**
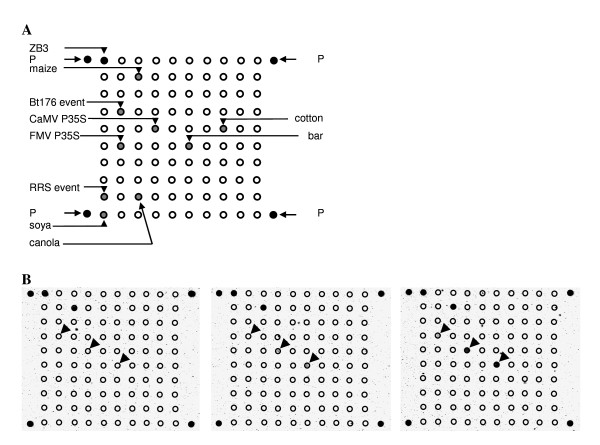
**Microarray with the schematic position of the different padlock ZIP-codes (A) and hybridization results of a dilution series (0, 0.1 and 0.5%) of maize Bt176 (B)**. GMO maize was diluted in wild type maize. The expected hybridisation signal for maize (endogenous zein) is always present while CaMV P35S and bar (in 0.1%) and Bt176 event (0.5%) emerge when the concentration increases. Arrows indicate the appearance of the Bt176 event, CaMV P35S and *bar *spots.

A dilution range of Bt176 maize was used for determining the sensitivity of probes for the CaMV 35S promoter (element), Bt176 (event) and the *bar *gene (element). Likewise, a RRS dilution series was used for the sensitivity of the probes for CaMV P35S and the RRS event and a MON1445 series for the CaMV P35S and the FMV P35S. The probes for CaMV P35S and the *bar *elements showed the highest sensitivity (to 0.1%), while the probes for RRS event and FMV P35S showed the lowest sensitivity (5%). Sensitivity was also tested against all other samples expected to be negative, so the Bt176 event probe was not only tested in a 0% Bt176 reference sample, but also in RRS and MON1445 samples and the wild-type mix that were assumed to be 0% Bt176 samples. In these experiments the padlock probes showed the same sensitivity, except for the *bar *probe that showed a slightly lower sensitivity (0.5% instead of 0.1%) when all 'negative' samples were included in the analysis. A typical series of arrays is shown in Figure 3, where the 0%, 0.1% and 0.5% Bt176 samples illustrate the different sensitivities for the CaMV P35S, *bar *and Bt176-event probes. A comparison of the CaMV P35S signals in different matrices showed a sensitivity of 0.1% in Bt176 and RRS while this was 0.5% in MON1445 material.

All plant species-specific probes (except for the maize zein probe) showed high specificity in these experiments. In some cases false positive spots were observed, but with significantly lower signals (usually over 100-fold lower, results not shown). This aspect will need to be further investigated when threshold levels for positive signals are going to be established.

### Performance in multiplex GMO experiments

**Figure 4:**
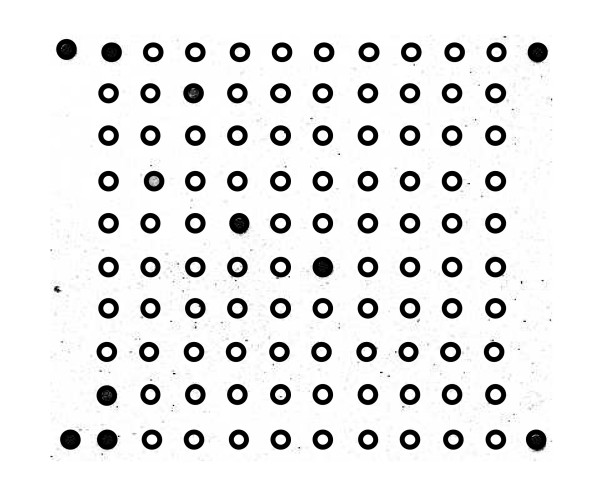
**Tenplex reaction with complex genomic GMO DNA**. 2.5% Bt176 maize and 2.5% RR soya in a tenplex with target DNA for seven padlock probes. Three padlock probes without target remain negative. For positioning of the spots, please consult Figure 3A.

The probe mixture was also tested in more complex samples (Table 3). In this multiplex setting all padlock probes performed as expected in the PPLMD approach. The sensitivity was at least 1% for CaMV P35S (in a mixture of 1% RRS; 1% Bt176; 1% MON1445; canola) and 1.7% for the GMO elements FMV P35S and *bar *(in a mixture of 1.7% RRS; 1.7% Bt176; 1.7% MON1445). The sensitivity of the event specific probes for RRS and Bt176 was 2.5% (in a mixture of 2.5% RRS; 2.5% Bt176) (Figure 4). In all experiments, ten padlocks were included. In the 2.5% mixture, the padlock probes were able to circularise on all the seven targets present, i.e. CaMV P35S, *bar*, Bt176 event, RRS event, soya-specific, maize-specific and the SpikeLock. The three additional padlocks were cotton-specific, canola-specific and FMV P35S. In one experiment where 25% of each GMO was present (100% RRS, Bt176 and MON1445, completed with canola) all ten padlocks were able to recognise their target and showed a positive signal on the microarray (data not shown).

In the non-target DNA sample (wild type canola + rice + sugar beet + potato: 25% each), only the SpikeLock and the canola signal were detected. No signal from the GMO-specific padlocks emerged above the background level. The species-specific padlock for maize showed some background in negative samples.

### Quantitative response

For the probes that showed signals significantly different from 0% in at least three consecutive dilution steps, the quantitativeness of the signals was evaluated. The input amount was plotted against the end signal and a trend line calculated. Ideally, both the slope and the correlation coefficient (R^2^) of the trend line would be 1. For these 5 tests (MON1445:CaMV P35S, Bt176:CaMV P35S, Bt176:*bar*, Bt176:event, RRS:CaMV P35S) the average slope and R^2 ^were 1.0210 +/- 0.1363 and 0.9756 +/- 0.0347 respectively, underlining the quantitative potential of this approach (results not shown).

## Discussion

The maintenance of (EU) GMO regulations in the light of increasingly complex worldwide logistic networks requires sensitive multimethods for the detection and identification of approved as well as unapproved GMO crop varieties, including unknown GMOs (Figure 1B).

So far a number of different approaches to develop such methods have been documented in the scientific literature [7,10,18,34,35], but sensitivity in a genomic DNA environment, lack of specificity, and high background levels are often reported as aspects that hamper the practical applicability of the different approaches. One of the most promising strategies in this area is the combination of probe ligation, amplification and microarray detection. The major advantage of this approach is that only the selected genomic sequences (employed in the padlock probe) are amplified and subsequently detected. The PPLMD system as described here is further developed and optimized with the aim to detect GMOs and GMO elements in a multiplex setting. The PPLMD approach has been developed as a screening method, but the resulting data do suggest that quantification may also be feasible. This aspect will, however, need further testing.

Optimisation of the protocol was reached primarily by three adjustments in comparison with earlier published PPLMD-like protocols [17,21,23,36,37]: the use of Vent^® ^exo^- ^DNA polymerase instead of HotStar Taq DNA polymerase, the use of a SpikeTarget and associated SpikeLock padlock probe, and by applying an improved LATE-PCR strategy [38] for the amplification phase.

In earlier experiments, HotStar Taq DNA polymerase was used to amplify the circularised padlock molecules. Here, 5'-3' exonuclease activity will degrade the previously transcribed circular single-stranded padlock molecule, probably resulting in fewer targets for PCR amplification. By experimenting with Vent^® ^exo^- ^DNA polymerase lacking this 5'-3' exonuclease activity, a strand displacement strategy of a circular template should allow the linear amplification of the circular padlock molecule, in a rolling circle-like amplification that increases the amount of target prior to PCR amplification. Although the results only showed a tendency that positive signals were improved and background signals were decreased compared to HotStar Taq DNA polymerase, the lack of exonuclease activity made Vent^® ^exo^- ^DNA polymerase the preferred choice to amplify circular molecules.

The second improvement relates to the introduction of the SpikeLock padlock probe and SpikeTarget molecules in the ligation reaction. The use of the SpikeLock padlock probe in combination with the SpikeTarget allows a basal reaction that serves as a ligation control, as well as visualisation of the positioning spots on the array that flank the ZIP-spots. Furthermore, the amount of SpikeLock (containing the cZIP-P) and SpikeTarget is equal in all samples and was thus used as an internal standard. Ligated molecules were amplified using the Cy3-labelled reverse primer and Cy5-labelled cZIP-P was added in all reactions. The calculated ratio is a measure for performance of the ligation reaction and this was also used to normalise the ratios of Cy3/Cy5. Additionally, Cy3- and Cy5-labelled cZIPB3 were added to the hybridization mixture to allow comparison between the arrays.

Finally, the PCR reaction for amplification of the circularised padlocks was performed on the basis of the so-called Linear After The Exponential (LATE) principle [38]. The LATE principle requires unequal amounts of forward and reverse primer. The Cy3-labelled reverse primer is present in a 500 nM concentration, while the forward primer is present in a 50 nM concentration. The forward primer is extended with two nucleotides to compensate for loss in T_m _due to the lower concentration. After the forward primer is depleted in the exponential amplification step, a linear phase is commenced in which preferentially the Cy3-labelled primer is extended, thus increasing the signal compared to an asymmetrical PCR (as performed by [23] where only the primer concentrations differed. Furthermore, to facilitate the LATE principle, the number of amplification cycles was increased from 40 to 80.

Using this optimised PPLMD protocol, experiments were performed in simplex and in multiplex. To establish the minimal amount that can be discriminated from a background signal, a dilution range with three GMOs was tested in the PPLMD system. As described in Table 3, low detection limits were achieved for CaMV P35S (recognising the 35S promoter that is often used in GMOs) and other targets in different GMOs tested.

The difference in sensitivity for CaMV 35S promoter sequence in the different GMOs can be explained by the number of integrations of the construct and the different genome sizes and ploidy numbers. DNA (200 ng) contains a limited amount of copy numbers of a certain target. In mixtures, the amount of each target is proportionally lower. The sensitivity for CaMV P35S was 0.1 (Bt176), 0.1 (RRS) and 0.5% (MON1445). RRS comprises the complete integration of the RRS genetic construct as well as two partial integrations [26]. Bt176 contains at least a double integration of the *bar *plasmid with the CaMV P35S [39]. MON1445 contains a single copy of both the CaMV and FMV 35S promoter sequences [26]. The calculated amount of CaMV target molecules in 200 ng 0.1% Bt176, RRS and MON1445 is 146, 176 and 62, respectively. These data suggest that more MON1445 is necessary to detect CaMV P35S compared to RRS and Bt176, which may explain the observed difference in sensitivity. Overall, these experiments show the sensitivity and applicability of the PPLMD approach in different percentages of a single GMO: most padlocks detect their target genomic sequence in the range of 0.1–0.5% GMO.

When DNA of 5% Bt176 and RRS was combined to yield 2.5% GMO each, the results showed that both GMO event targets could be detected. In a more complex sample of 1.7% of each Bt176, RRS and MON1445, the GMO elements FMV P35S and *bar *could still be detected, while the GMO element CaMV P35S, often used as a screening element, was still detectable in the lowest GMO mixture tested (1%). The sensitivity of the RRS-event was 5% in a pure sample and 2.5% in a mixture. This may be explained by the fact that in the dilution series, no 2.5%-step was included: the sensitivity of 5% in a pure sample may therefore be an underestimation. Also, in a non-target DNA mixture, padlocks (except for the maize padlock) of which the genomic target was not present did not result in a visible and significant signal on the microarray. Currently, alternative padlock probes are being investigated for the maize *hmg *and *adh *genes. The positive control for canola (and the SpikeLock for that matter) gave the expected positive signal. In follow-up experiments, padlock concentrations can be adjusted in the PPLMD protocol to further optimise the sensitivity of the approach. In mixtures of GM crop species the sensitivity was lower compared to the simplex experiments. Species-specific signals were lower because the change of 100% species-specific target in a pure sample, to 33% in a more complex mixture. An explanation for the overall reduced sensitivity could be a matrix effect in which non-DNA factors of the combined DNA samples influence the padlock ligation reaction as part of the PPLMD, thereby reducing the signal that would have been reached when the samples were analysed separately. Nevertheless all selected elements could be detected down to at least 2.5% and in single cases down to 1%. Here further probe selection and optimisation and standardisation of the PPLMD reaction conditions may lead to further improvement of the sensitivity in these 'real life' samples.

The outcome of this study demonstrates the applicability of the PPLMD method for detection of known and approved GM crop events. In previous publications authors often make use of synthetic target molecules when analysing GMOs in simplex or mixtures. In this article it was demonstrated that the sensitivity levels in real-life samples are between 0.1 and 5% in single DNA samples and between 1 and 2.5% in more complex mixtures of plant DNA isolated from seed. The negative controls are non-target plant species in equimolar amounts that reflect a real-life situation, instead of non-target- water-controls. The PPLMD method has been optimised using Vent^® ^exo^- ^DNA polymerase in combination with a SpikeLock padlock probe to increase the positive signals and reduce the background levels. From the results it can be concluded that padlock probe detection using PCR in combination with microarray detection allows positive detection of GMOs and GMO elements in plant tissues. In this article, a proof of principle was demonstrated to show the feasibility, sensitivity and applicability of PPLMD-based detection. PPLMD can thus be used as a screening method: detected approved GMOs can subsequently be further quantified by EU-validated event-specific quantitative PCRs. The method also offers a good basis for the detection of non-authorised GMOs (NAGs), including unknown GMOs. This latter category may gain further importance with the current increase of new GMO crop varieties on the world market, the potential of modern breeding strategies to adjust the physiology of crops and the growing complexity of global logistic networks.

## Authors' contributions

TWP, HGB, AMAH and MMV carried out the design and testing of padlock probes and the molecular analyses. TWP drafted the manuscript. JPD participated in the design of the study and performed the data analysis with HGB. CDS contributed with knowledge on the padlock probe system and helped to draft the manuscript. EJK, JPD and HJMA initiated the study, participated in its design and coordination and helped to draft the manuscript. All authors read and approved the final manuscript.
